# Emergence and predominance of GII.17[P17] noroviruses in Brazil, 2023–2024

**DOI:** 10.1038/s41598-026-49730-6

**Published:** 2026-04-25

**Authors:** Fábio Correia Malta, Mateus de Souza Mello, Ighor Arantes, Juliana da Silva Ribeiro de Andrade, Alexandre Madi Fialho, Gabriel Assad Baduy, Rafael Brandão Varella, Maria Angelica Arpon Marandino Guimarães, Fernanda Marcicano Burlandy, Tulio Machado Fumian

**Affiliations:** 1 Laboratory of Comparative and Environmental Virology, Oswaldo Cruz Institute, Oswaldo Cruz Foundation, Rio de Janeiro, RJ 21045-900 Brazil; 2 Department of Infectious and Parasitic Diseases, School of Medicine, Clementino Fraga Filho University Hospital, Federal University of Rio de Janeiro, Rio de Janeiro, 21941-590 Brazil; 3 Department of Microbiology and Parasitology, Institute Biomedical, Federal Fluminense University, Niterói, 24210-130 Brazil; 4 Laboratory of Arboviruses and Hemorrhagic Viruses, Oswaldo Cruz Institute, Fiocruz, Rio de Janeiro, Brazil

**Keywords:** Diseases, Gastroenterology, Genetics, Medical research, Microbiology

## Abstract

Norovirus is a leading cause of acute gastroenteritis (AGE) worldwide, affecting all age groups. In the present study, we investigated fecal samples from medically attended AGE patients received from ten Brazilian states collected during 2023 and 2024. Norovirus GI and GII were detected and quantified using RT-qPCR, and norovirus-positive samples underwent genotyping through sequencing the ORF1/2 junction region. During the two-year period, norovirus prevalence was 22.5%. GII genotypes were predominant, accounting for 85.6% of the genotyped samples. By genotyping, we detected the circulation of 19 norovirus genotypes, of which the emergence of GII.17[P17] in 2023 and its predominance in 2024. Comparison of cycle threshold (Ct) values indicated that the median viral load was significantly higher for norovirus GII.17 compared to GII.4 (20.9 and 22.3, respectively (*p* = 0.0493). Additionally, we showed that GII.17 patients were older compared to GII.4 patients. Our study is the first in South America to demonstrate replacement of the previous dominant genotype (GII.4 Sydney[P16]) to GII.17[P17] in 2024, currently disseminated worldwide.

**Supplementary Information:**

The online version contains supplementary material available at 10.1038/s41598-026-49730-6.

## Introduction

Noroviruses are the leading cause of acute gastroenteritis (AGE) across all age groups globally, accounting for nearly 20% of AGE cases and an estimated 200,000 deaths annually, primarily in low- and middle-income countries^1–4^. Following the widespread introduction of rotavirus vaccines, noroviruses have become the main viral cause of pediatric AGE in many regions^5–7^.

Noroviruses are non-enveloped, positive-sense, single-stranded RNA viruses of the genus *Norovirus*, within the *Caliciviridae* family. Their ~7.5 kb genome is organized into three open reading frames (ORFs): ORF1 encodes the nonstructural proteins, including the RNA-dependent RNA polymerase (RdRp); ORF2 encodes the major capsid protein VP1; and ORF3 encodes the minor structural protein VP2. Frequent recombination events at the ORF1–ORF2 junction result in the emergence of new strains that combine distinct polymerase and capsid genotypes^8,9^. Based on VP1 diversity, noroviruses are classified into at least ten genogroups (GI–GX) and 49 genotypes, of which five (GI, GII, GIV, GVIII, and GIX) infect humans^10,11^. Among human noroviruses, GII viruses cause most outbreaks and sporadic infections worldwide, with GII.4 variants predominating for nearly three decades^12–14^. The periodic emergence of novel GII.4 variants, such as Grimsby 1995, Farmington Hills 2002, New Orleans 2009, and Sydney 2012, has been linked to amino acid substitutions in key antigenic epitopes within the subdomain P2, the most exposed region of the VP1 protein, enabling immune escape and rapid global spread^15,16^. Nevertheless, non-GII.4 genotypes such as GII.2, GII.6, and GII.17 have periodically become dominant in specific regions or seasons^17–19^, illustrating the evolutionary plasticity of noroviruses.

The GII.17 genotype has drawn global attention due to its emergence and capacity for extensive geographic spread. After decades of sporadic detection, the GII.17[P17] variant known as “Kawasaki 308” caused widespread outbreaks in Asia during 2014 and 2015 and was subsequently reported in multiple continents^20,21^. More recently, a resurgence of GII.17[P17] activity was reported across Europe and North America, coinciding with a sharp decline in GII.4 Sydney[P16] detections^22,23^. During the 2024-2025 season, GII.17 accounted for up to 75% of norovirus outbreaks in the United States and between 17% and 64% of all GII detections in several European countries^22,23^. Phylogenetic analyses revealed that most of these strains were closely related to the GII.17[P17] “Romania 2021” lineage and that two new sub-lineages within the RdRp region had emerged^22,24^.

In Brazil, noroviruses have been consistently detected as a major cause of AGE in children, displaying extensive genotype diversity in both outbreak and sporadic cases^25–30^. To date, a large proportion of norovirus infections in Brazil has been attributed to GII.4 lineages, but recent findings elsewhere raise the possibility of a similar genotype transition occurring in South America^27,28,30–33^.

In the present study, we report the emergence and dominance of GII.17[P17] noroviruses in Brazil from January 2023 to December 2024. Through molecular detection and genetic characterization of norovirus in stool samples from medically attended patients with AGE, we investigated its epidemiology, prevalence, and genetic diversity of circulating strains. This study provides follow-up surveillance of norovirus genotypes circulating in Brazil and presents the first evidence of sustained circulation of GII.17[P17] in the country, contributing to a broader understanding of the ongoing global re-expansion of this genotype.

## Results

### Norovirus epidemiology

**Fig. 1:**
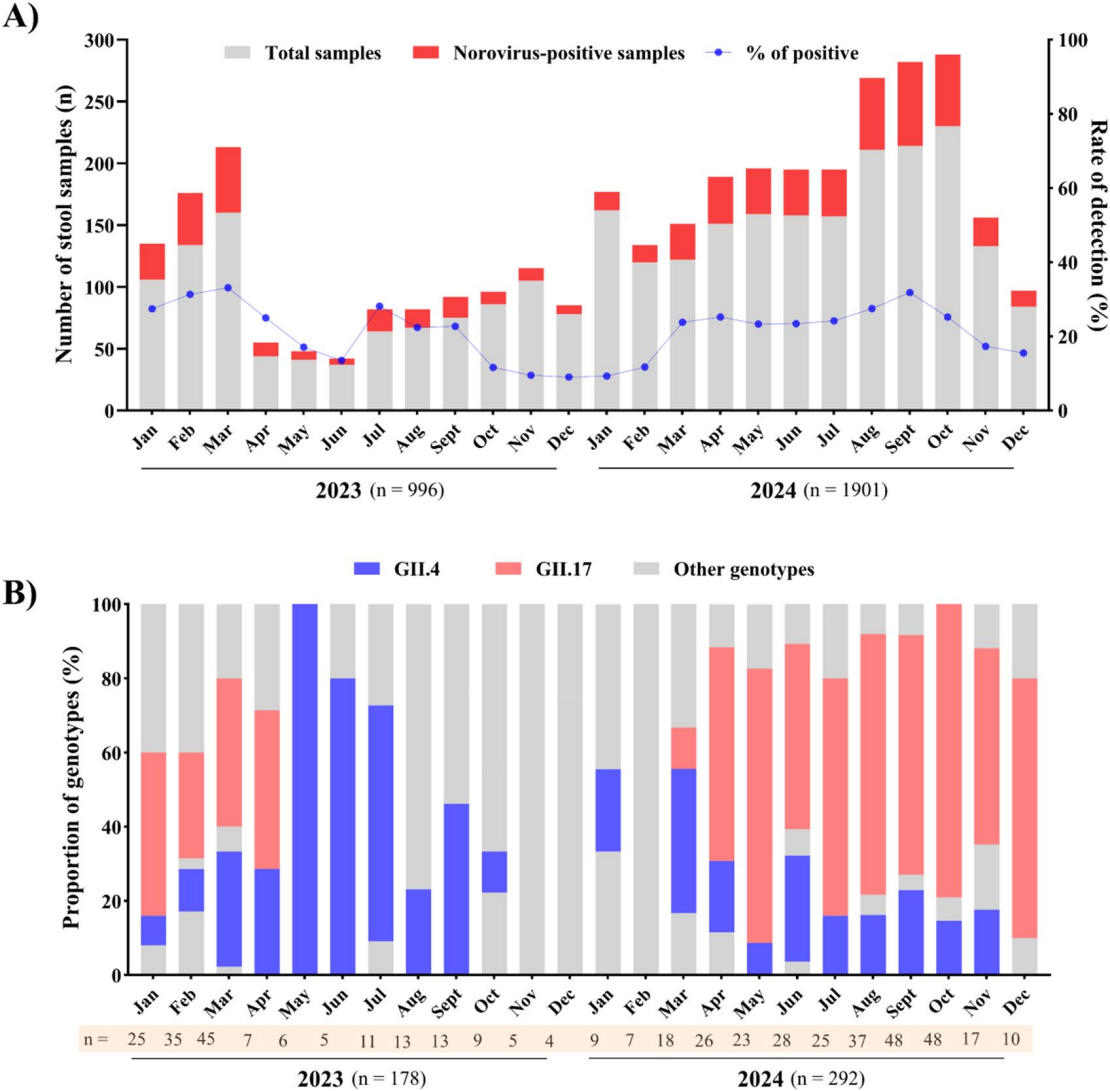
(**A**) Monthly distribution of tested acute gastroenteritis samples, norovirus positive samples, and detection rates in Brazil, 2023–2024. The total number of samples analyzed is represented in gray, the total number of norovirus-positive samples in red, and blue line represents the norovirus detection rate. (**B**) Monthly distribution of genotypes during surveillance period in Brazil, 2023–2024. The total number of noroviruses GII.4 is represented in blue, GII.17 in red, and the other genotypes in gray.

During the two-year study period, we analyzed a total of 2897 stool samples from medically attended patients with AGE from 10 Brazilian states, covering Southern, Southeastern and Northeastern regions. Norovirus was detected in 22.5% of samples (n = 651), being 22.5% (224/996) in 2023 and 22.5% (427/1901) in 2024. The highest prevalence of norovirus was found in the Southern region with 33.4% (308/923), followed by Southeastern region with 20.3% (207/1,020), then Northeastern with 14.3% (136/954) (Table 1). Norovirus was detected year-round without marked seasonality. Regarding monthly detection rates, we observed the lowest and highest norovirus positivity in December (9%) and March 2023 (33.1%), respectively (Fig. 1A). In 2024, we observed a significant increase in AGE cases, receiving nearly twice the number of samples as the previous year. Despite this surge, the proportion of norovirus infections among the samples received remained identical in both years (Fig. 1A).

**Table 1:** Prevalence of norovirus infection in medically attended patients with acute gastroenteritis by region in Brazil, during 2023–2024. ^1^*p*-values were calculated comparing norovirus frequency of detection in Southern with each other regions.

Region	No. of fecal samples—positive/tested (%)			*p*-value^1^	*χ*^*2*^ Value
	2023	2024	Total		
Northeastern	29/220(13.2)	107/734 (14.6)	136/954 (14.3)	< 0.0001	94.90
Southeastern	69/350 (19.7)	138/670 (20.6)	207/1,020 (20.3)	< 0.0001	42.53
Southern	126/426 (29.6)	182/497 (36.6)	308/923 (33.4)	–	–
Total	224/996 (22.5)	427/1,901 (22.5)	651/2,897 (22.5)	–	–

Norovirus was detected across all age groups, the median age was 45 months in 2023 and 34 months in 2024, with detection rates varying from 27.7% (132/476) in adults aged between 21 and 50 years old to 13% (44/338) in children up to six months (Table 2). Similar norovirus detection rates were found among children aged 7 to 24 months and patients ≥6 years old (children, adolescents, adults and elderly), without statistical significance (*p* > 0.05). As for gender, norovirus detection rates were identical between females (22.5%; 319/1419) and males (22.5%; 333/1478).

**Table 2:** Distribution of norovirus infection in different age groups of medically attended patients with acute gastroenteritis in Brazil, during 2023–2024. *m* months old, *y* years old. ^1^*p*-values were calculated between the age group of 21–50y and each other.

Age group	No. of fecal samples—positive/tested (%)			*p*-value^1^	*χ*^*2*^ Value
	2023	2024	Total		
0–6 m	14/86 (16.3)	30/252 (11.9)	44/338 (13)	< 0.0001	25.09
7–12 m	33/140 (23.6)	72/265 (27.2)	105/405 (25.9)	0.5471	0.3402
13–24 m	36/182 (19.8)	98/346 (28.3)	134/528 (25.4)	0.3990	0.6777
25 m–5y	38/199 (19.1)	70/418 (16.7)	108/617 (17.5)	< 0.0001	15.68
6y–20y	22/80 (27.5)	52/225 (23.1)	74/305 (24.3)	0.2832	1.115
21y–50y	57/220 (25.9)	75/256 (29.3)	132/476 (27.7)	–	–
> 51y	24/89 (27)	30/136 (22.1)	54/225 (24)	0.2962	1.059

Following descriptive analyses, we explored the relationship between demographic and contextual factors and the distribution of circulating norovirus genotypes using a multiple correspondence analysis (MCA). By integrating patient age range, geographic region, study period, sex, and viral genotype, the MCA factor maps revealed patterns of association between these variables, suggesting that certain genotypes tend to occur more frequently in specific epidemiological contexts, such as age ranges or regions (Figs. S1A and S1B). The analysis of the contribution of the variables showed that age range and geographic region were the main contributors to the first dimension, while study period and sex contributed more strongly to the second dimension (Figs. S1C and S1D). Permutation tests indicated that the observed inertia for both dimensions was significantly greater than expected under random conditions, corroborating the robustness of the associations identified in the MCA (Figs. S1E and S1F). To further quantify these relationships, logistic regression models were applied to assess the factors associated with infection by the GII.17 genotype. Both univariate and multivariate models indicated that the variables studied contributed to explaining the distribution of this genotype, although the magnitude of the associations differed after adjusting for potential confounding factors (Fig. S2). Consistent, these analyses suggest that demographic and contextual factors, including age group, geographic region, study period, and sex, are associated with the circulation patterns of norovirus genotypes in Brazil.

### Norovirus molecular characterization—emergence of GII.17 in 2023 and predominance in 2024

**Fig. 2:**
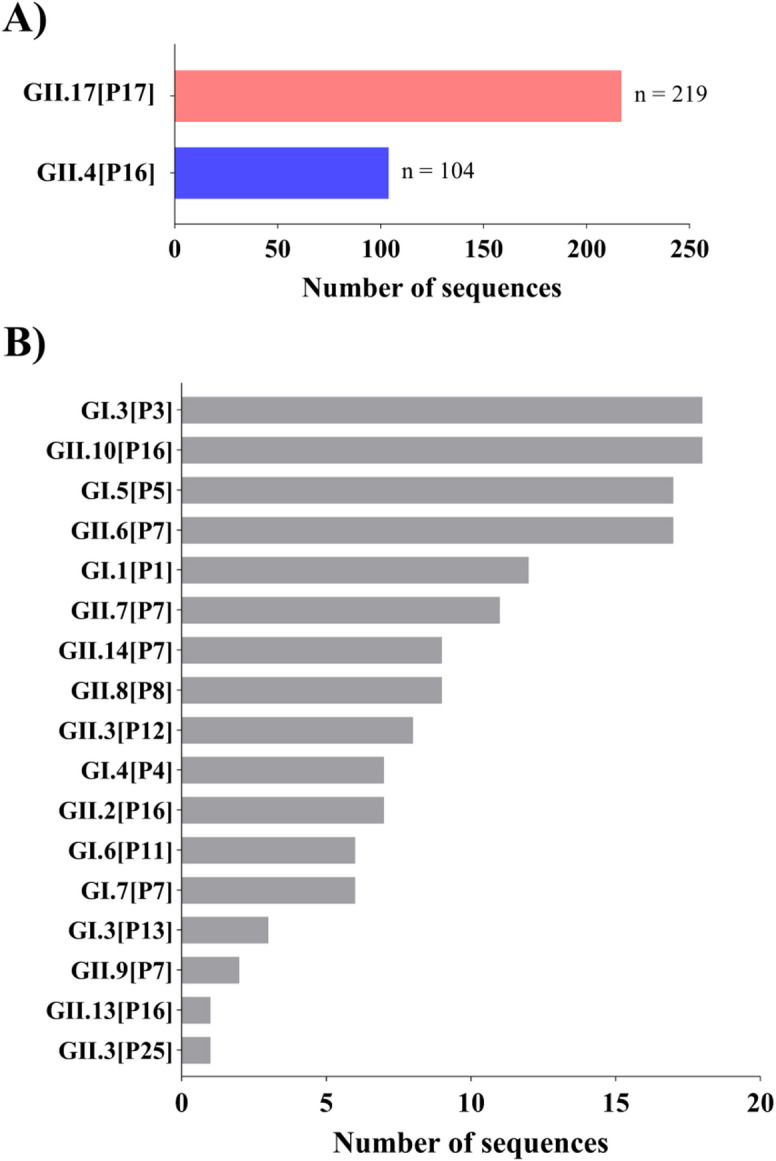
(**A**) Norovirus GII.4[P16] and GII.17[P17] identified during surveillance period 2023–2024. (**B**) Distribution of norovirus P-types and VP1 genotypes identified during the study period.

**Fig. 3:**
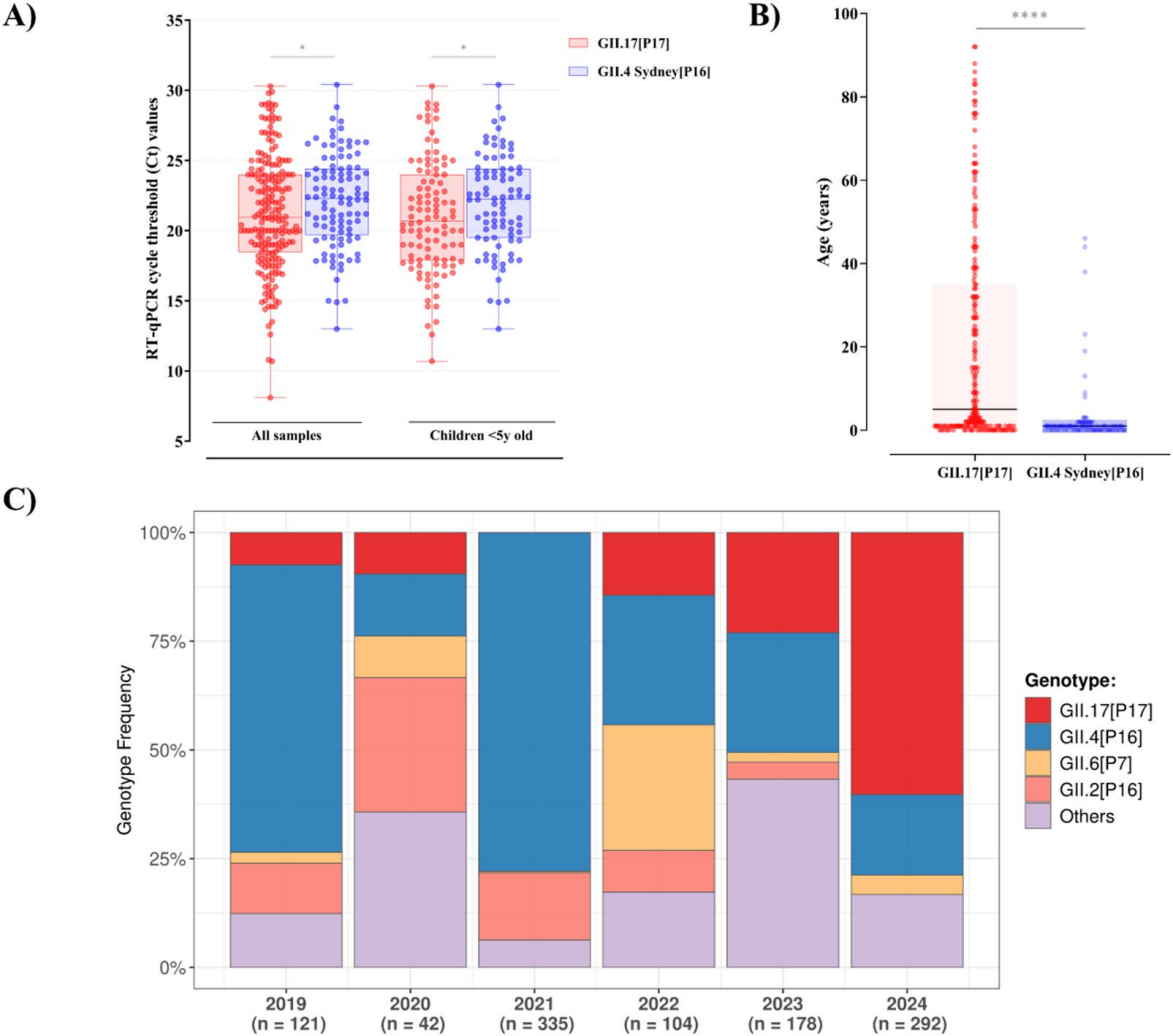
(**A**) Norovirus GII.4[P16] and GII.17[P17] viral load by cycle threshold value (CT) among different age groups in Brazil, 2023–2024. (**B**) Age distribution of noroviruses GII.4[P16] and GII.17[P17]. (**C**) Shift of noroviruses genotypes frequency among 2019–2024. *p ≤ 0.05,****p ≤ 0.0001. The 2019–2022 data used to draw Fig. 3C were taken from the paper by Sarmento et al. 2023^30^.

Among the 651 norovirus-positive samples, we successfully sequenced 72.2% (n = 470), being 178 (79.5%) from 2023 and 292 (68.4%) from 2024. Overall, norovirus GII predominated and was detected in 85.6% (n = 404) of samples; GI was detected in 12.6% of samples and co-detection of GI and GII in 1.8%. The dual genotyping of polymerase and capsid regions showed the circulation of 19 different norovirus genotypes. During the two-year period, GII.17[P17] was the predominant genotype found in 46.6% (219/470) of samples, and the second most common genotype was GII.4 Sydney[P16], with detection rate of 22.1% (104/470) (Fig. 2A). When comparing our current findings (2023–2024) with those previously reported by Sarmento et al.^30^ (2019–2022), we observed a shift in norovirus genotype circulation over the 2019–2024 period, characterized by the replacement of GII.4. While GII.17[P17] and GII.4 Sydney[P16] circulated at comparable levels in 2023 (24.2% and 27.5%, respectively), GII.17[P17] emerged as the dominant strain in 2024, being detected in 60.3% (176/292) of samples compared to 18.8% (55/292) for GII.4 Sydney[P16] (Fig. 2B). In addition to these two predominant genotypes, 17 others were detected at lower frequencies. The genotypes GII.10[P16] and GI.3[P3] were detected in 3.8% (18/470) of samples each, and genotypes GII.6[P7] and GI.5[P5] were detected in 3.6% (17/470) each. The other genotypes were detected in less than 3% of genotyped samples (Fig. 2B). Furthermore, we observed a replacement in circulating genotypes between 2023 and 2024, when compared to the surveillance study performed by Sarmento et al. 2023 between 2019 and 2022 (Fig. 3C)^30^.

We also compared Ct values (used as a proxy of viral load) between GII.17- and GII.4-positive samples to assess differences in viral load profiles. We found Ct values were significantly lower in GII.17, indicating a higher viral load compared with GII.4 samples. The median Ct values of GII.17 and GII.4 samples were 20.9 and 22.3, respectively (*p* = 0.0493). Amongst young children (<5 years), median Ct values were 20.7 for GII.17 samples and 22.3 for GII.4 samples (*p* = 0.0419) (Fig 3A). For patients older than five years, comparisons were not possible due to the limited number of GII.4-positive samples detected in this age group.

With regards patients infected with GII.17[P17] or GII.4 Sydney[P16], we analyzed the age distribution and observed that among patients infected with GII.17[P17] the median age was 5 years old, and for GII.4 Sydney[P16] the median age was 12 months. Interestingly, among the 219 samples characterized as GII.17, 111 (50.7%) were from patients younger than five years old and 108 (49.3%) were from patients older than five years old. For GII.4-infected patients, among the 104 samples, 92.3% (n = 96) were from children less than five years old, and 7.7% (n = 8) were from patients older than five years. Moreover, GII.17-infected patients were significantly older compared to GII.4-infected patients (*p* < 0.0001) (Fig 3B). By age group, among GII.17 cases, the proportion of children (0–5 years), older children, adolescents and adults (6–65 years), and older adults (>65 years) were 50.7% (111/219), 41.1% (90/219), and 8.2% (18/219), respectively. In contrast, among GII.4 cases, a higher proportion occurred in children aged 0-5 years (92.3%; 96/104), compared with patients aged 6-65 years (7.7%; 8/104); no GII.4 infections were detected among older adults (>65 years). The oldest patients infected with GII.17 and GII.4 were 92 and 46 years of age, respectively. It is worth noting that norovirus detections in older adults (aged >65 years) were exclusively associated with GII.17 cases.

### Phylogenetic analysis

**Fig. 4:**
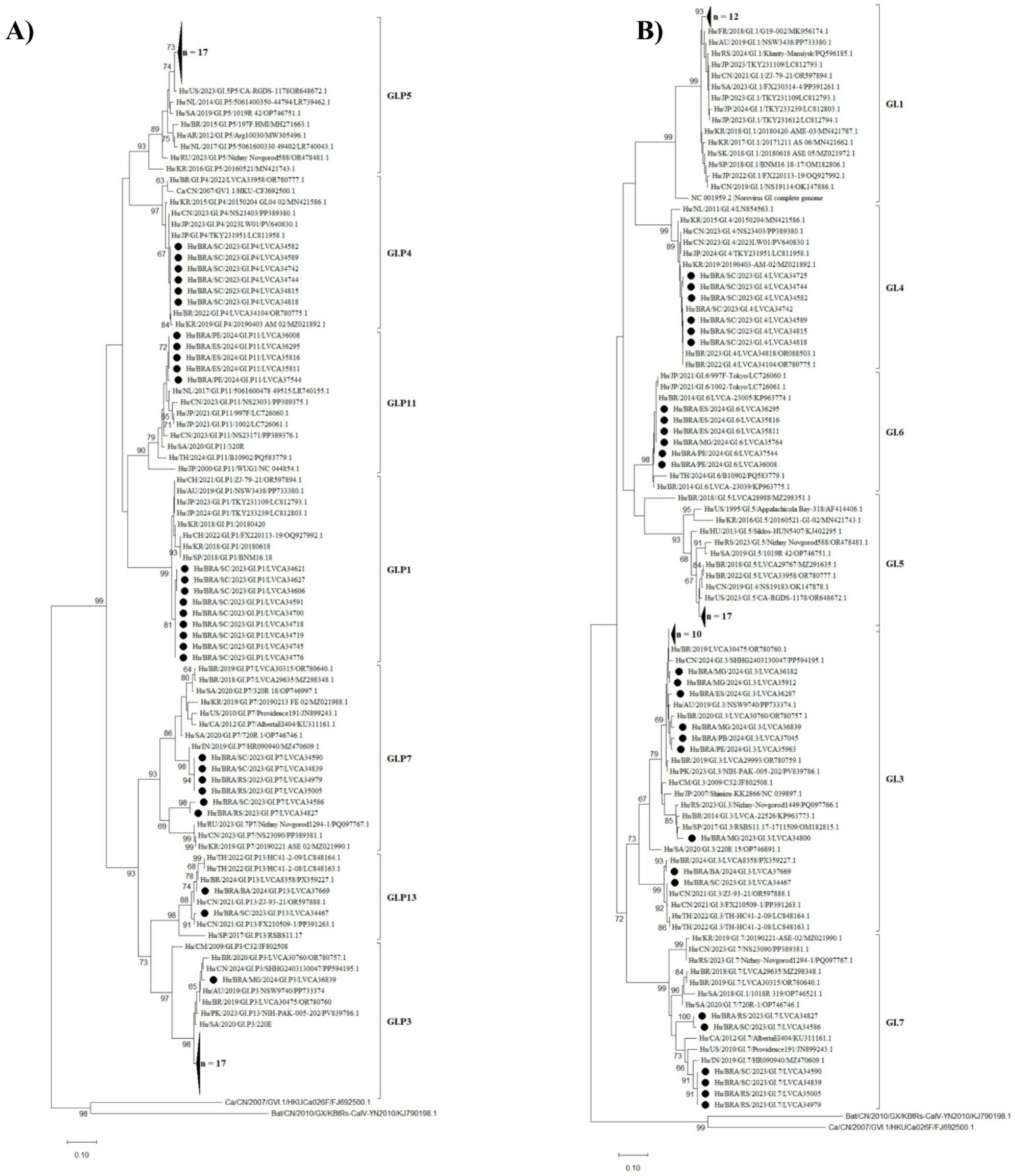
Phylogenetic analyses based on RNA-dependent RNA polymerase (RdRp) (**A**) and Major Viral Capsid Protein (VP1) (**B**) nucleotide (nt) sequences of GI norovirus Brazilian strains. Strains obtained (marked with a black circle) are shown as per host followed by country followed, State, year, genotype and LVCA internal register number (i.e., Hu/BRA/RS/2023/GI.7/LVCA34979). The neighbor-joining phylogenetic tree was constructed with bootstrap tests (2000 replicates), based on the Kimura two-parameter mode. Bootstrap values above 60% are given at branch nodes.

**Fig. 5:**
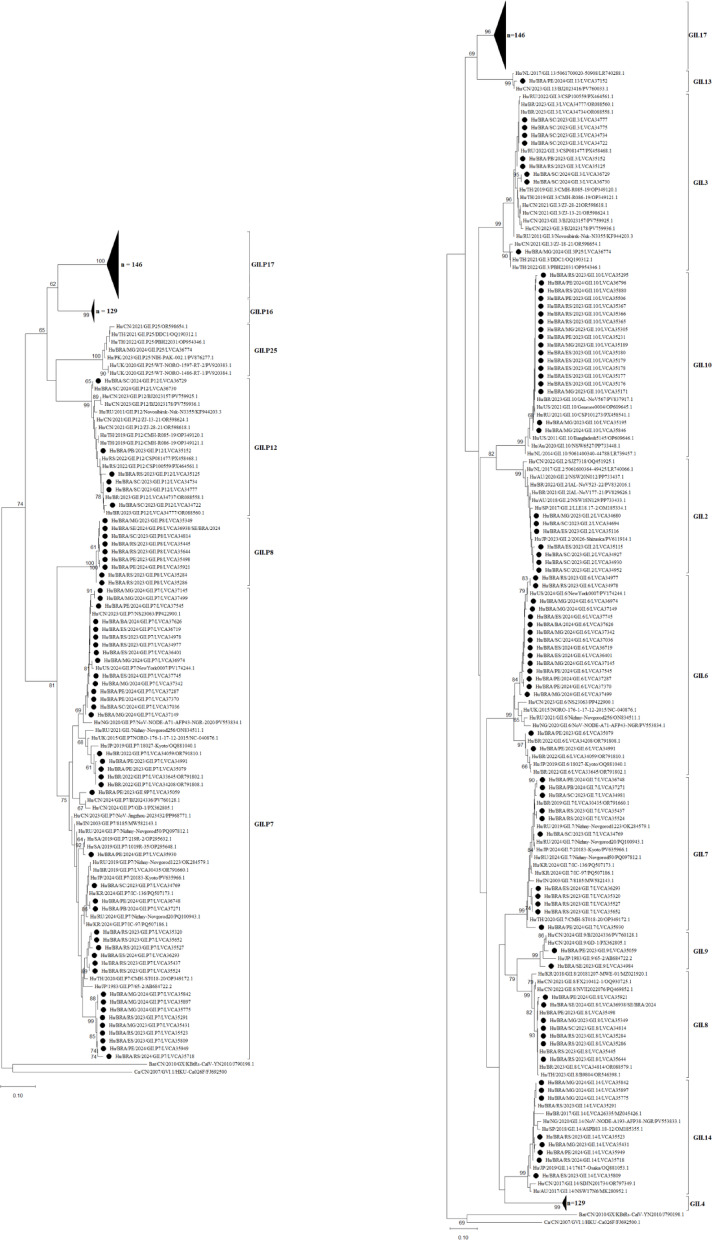
Phylogenetic analyses based on RdRp (**A**) and VP1 (**B**) nucleotide (nt) sequences of GII norovirus Brazilian strains. Strains obtained (marked with a black circle) are shown as per host followed by country followed, State, year, genotype and LVCA internal register number (i.e., Hu/BRA/RS/2023/GII.10/LVCA35177). The neighbor-joining phylogenetic tree was constructed with bootstrap tests (2000 replicates), based on the Kimura two-parameter mode. Bootstrap values above 60% are given at branch nodes.

The partial RdRp nucleotide sequences (~250 bp) of noroviruses strains were phylogenetically analyzed together with the reference strains as shown in Figs. 4A and 5A. With regards to GII.P17 pol type, Brazilian sequences shared between 95.2 and 100% of nucleotide identity (majority exhibited >99%) and were exclusively associated with the GII.17 capsid genotype. Comparative analysis with global reference sequences showed that GII.P17 strains detected in this study were closely related to strains reported previously from Argentina (KX061540), Australia (KT285173), Brazil (KR074151 and KR074152) Cameroon (KJ946403 and JF802505-07), China (KU557788-90 and KT633384), Japan (LC486747 and LC486752), Russia (KY210932, KY210937, and KY210939), South Korea (KU561250 and KU561251), Taiwan (KJ156329) and Thailand (LC848162), with nucleotide sequence identities ranging from 95.5 to 100% (Fig. S1A). The second most predominant pol type identified was GII.P16 and was detected in association with multiple capsid VP1 genotypes, specifically GII.2, GII.4, GII.10, and GII.13. Brazilian GII.P16 sequences obtained in this study exhibited nucleotide identity ranging from 92.8 to 100%. Compared to global GII.P16 strains, Brazilian P16 sequences were closely related to sequences from Argentina (MW305632), Australia (MG002630 and GQ845369), Brazil (PV837890 and PV837892), Canada (PQ031233 and PP661667), China (OQ451925 and PV759885), Japan (AB541325 and PV636038), Mexico (MZ478136), the Netherlands (LR740019 and LR740066), Russia (PV746275 and PV746276), South Korea (PQ507183), Spain (OM185334), Thailand (PP564831, and PP549882), and the United States (FJ537136, and PQ196524) (Fig. S3A).

With regards to GI types, GI.P3 and GI.P5 were the most frequently identified (Fig. 4A). Most GI.P3 sequences from this study (n = 18) formed a distinct, well-supported cluster (bootstrap 98%) with nucleotide identities ranging from 99.8 to 100%, and clustered with sequences from Brazil (OR780757 and OR780760), China (PP594195) and Pakistan (PV839786). Brazilian GI.P5 sequences from this study formed a single cluster and showed nucleotide identity varying from 99.6 to 100%. Comparing to previously reported sequences, Brazilian GI.P5 strains clustered with sequences from Argentina (MW305496), Brazil (MH271663), the Netherlands (LR739462 and LR740043), Russia (OR478481), South Africa (OP746751), South Korea (MN421743), and the United States (OR648672), with nucleotide identity ranging from 85.9 to 99.6%.

Among GII noroviruses, partial VP1 sequence analysis showed that GII.17 strains exhibited nucleotide identities ranging from 94.2 to 100%. Brazilian GII.17 sequences clustered with recently circulating strains from a newly identified cluster represented by the Romania/2021 strain (OP805364/446225/Romania/2021), which is phylogenetically related to the GII.17[P17] cluster C lineage (Kawasaki323/Japan/2014). These sequences were less genetically related to GII.17[P17] strains that rapidly spread across continents during 2014–2016, corresponding to cluster D (represented by Kawasaki308/Japan/2014) (Fig. S3B). Regarding GII.4 sequences identified in this study, all of them belonged to the Sydney 2012 variant and shared 96-100% of nucleotide identity with each other. High sequence identity was also observed with contemporaneous Sydney 2012 strains reported from Brazil (OR088520 and OQ891865), China (PV759885), India (MW362541), Japan (PV636038 and PV636058), Russia (PX458544 and PX458550), and the United States (PQ213435). Additionally, 17 other genotypes were detected at lower frequencies during the surveillance period (Fig. 5B).

Among GI noroviruses, genotypes GI.1, GI.3, and GI.5 were most frequently detected (Fig. 4B). GI.1 sequences (n = 12) showed high nucleotide identity among themselves (99.7-100%) and were genetically similar to sequences detected from Australia (PP733380), France (MK956174), Russia (PQ596185), Japan (LC812793, LC812803, and OQ927992), China (OR597894 and OK147886), South Africa (PP391261), South Korea (MN421787 and MN421662), and Spain (OM182806), with nucleotide identities varying from 97.2 to 98.1%. GI.3 sequences (n = 19) were closely related to strains from Australia (PP733374), Brazil (OR780757, OR780759, and PX359227), Cameroon (JF802508), China (PP391263, PP594195, and OR597888), Japan (NC039897), Pakistan (PV839786), Russia (PQ097766), South Africa (OP746891), and Thailand (LC848163). Brazilian GI.5 sequences showed high nucleotide identity (99.3-100%) and were genetically similar to reference strains from Brazil (MZ298351 and MZ291635), China (OK147878), Hungary (KJ402295), Russia (OR478481), South Korea (MN421743), South Africa (OP746751), and the United States (OR648672).

## Discussion

Norovirus is a leading cause of AGE globally and is associated with an estimated 200,000 deaths and a societal cost of approximately US$60 billion annually^3^. In this study, we report data on the positive rates of norovirus among medically attended AGE patients from ten Brazilian states, encompassing a population of roughly 100 million people, nearly half of the country’s population. In addition, we present an analysis of the virological and molecular epidemiological features of these infections. Overall, norovirus was detected in 22.5% of samples, and our findings demonstrate a clear shift in the predominant circulating genotypes over the two-year surveillance period.

Historically, norovirus GII.4 variants have been responsible for most of global AGE cases, typically accounting for more than half of all infections. This long-standing predominance has been repeatedly documented in molecular surveillance studies carried out worldwide^11,14,30,34–37^. Between 2014 and 2016, GII.17 viruses emerged in Asia, driven by the surge of two novel phylogenetic clusters (currently namely C and D), which displayed major changes in VP1 and a newly identified polymerase type (GII.P17)^20,38,39^. Differences in antigenicity, enhanced binding to histo-blood group antigens (HBGAs), epithelial cells-expressed carbohydrates that facilitate norovirus attachment and infection, and their ability to infect different populations likely contributed to their rapid spread across multiple continents^19,40–42^.

The shift in age distribution observed in this study, where norovirus infections during 2023–2024 were most prevalent among adults aged 21–50 years, likely reflects the epidemiological impact of the emergence and dominance of GII.17[P17] during the period. In Hong Kong, China, a similar pattern was reported during the 2014–2015 emergence of the GII.17 Kawasaki. Norovirus GII.17-infections occurred disproportionately among older children and adults (median age of 49 years) and a higher proportion of elderly cases ≥85 years, contrasting with the age distribution of GII.4 cases that predominantly affects young children (median age of 1 year)^20^. In Brazil, similar findings were reported during an AGE outbreak in Santa Catarina state, where GII.17[P17] infections occurred predominantly in adults (71.4% of GII.17 cases), whereas GII.4[P16] infections were detected exclusively in children^29^. Those findings further support the tendency of GII.17 to shift the burden of disease toward older age groups, typically less affected by GII.4-dominated circulation.

Regarding norovirus incidence and age-group distribution, we observed that in 2023 and 2024 the highest prevalence of norovirus infection (27.7%) occurred among adults aged 21–50 years. This pattern contrasts with findings from previous surveillance studies in Brazil conducted by our group between 2017 and 2022^28,30^, in which the highest detection rates were consistently observed in children aged 7–24 months. In this same line, several studies worldwide have reported that norovirus prevalence is typically highest in children aged 7–24 months compared to other age groups^43–47^.

In Brazil, the emergence of GII.17[P17] in 2023 and its dominance in during 2024, aligns with a broader global pattern of genotype GII.17 activity. First evidence of the recent re-emergence of GII.17[P17] was provided by the genome characterization of a GII.P17 strain associated with a large outbreak in Romania in 2021^24^. Further phylogenetic analyses of sequences from Nizhny Novgorod, Russia, demonstrated the spread of GII.17 subcluster C2 (Romania-2021 like) in 2021–2023^48^. In that study, authors also demonstrated that this lineage corresponds to a newly defined subcluster of GII.17 cluster C (subcluster C2), distinct from the Kawasaki-2014 subcluster (C1) that circulated during the mid-2010s.

Data from six European countries and the United States of America (USA) during the 2023-2024 season demonstrated a marked rise in GII.17 detections, with this genotype comprising 17–64% of all GII cases depending on the country, following by a decline in GII.4 circulation^22^. Most of the contemporary GII.17 strains in these regions clustered closely with the Romania-2021 lineage, and two new P17 sublineages were identified, suggesting further diversification and ongoing adaptive evolution. These findings, documented across multiple surveillance systems, indicate that GII.17 has reemerged after nearly a decade of relative quiescence and can achieve substantial dominance with antigenically novel GII.17 variants. Additionally, in the USA, GII.17 outbreaks increased from 7.5% of all outbreaks in 2022–2023 to 34% in 2023-2024, eventually reaching 75% of all norovirus outbreaks by early 2025, effectively replacing GII.4 as the predominant genotype^23^. Consistent with this trend, surveillance studies from both South Korea and Shanghai, China, showed a shift in genotype circulation, with GII.17 emerging and subsequently overtaking previously dominant GII.4 variants during late 2024. In South Korea, nationwide dual-typing data showed that GII.17[P17] increased sharply from November 2024 onward, rapidly displacing GII.4[P31] and GII.4[P16]^49^. A similar pattern was observed in Shanghai, where GII.17[P17] became the predominant genotype in 2024, and accounted for 43.4% of all typed strains. Those Chinese strains phylogenetically clustered with newly emerging GII.17[P17] lineages detected in USA in 2024 and 2025^50^.

In Brazil, the emergent GII.17[P17] lineage was first identified in pediatric inpatients with AGE from the Northern region in 2016^51^. However, retrospective analyses indicated that its introduction occurred earlier. In a previous study from our group using Bayesian analysis of full ORF2 sequences, it was estimated that GII.17 Kawasaki_2014 was likely introduced into Brazil around mid-2014, with Hong Kong identified as the most probable source of these introductions. Phylogenetic reconstruction further revealed at least four independent introduction events during 2014 that coincided with the FIFA World Cup held in the country in that year^51^. Over subsequent years, GII.17 continued to circulate at low levels across multiple Brazilian regions, as documented in nationwide surveillance datasets spanning 2017–2018^28^ and 2019–2022^30^, where it appeared sporadically among a broad diversity of co-circulating genotypes, including the major GII.4 Sydney[P16], GII.2[P16], and GII.6[P7]. These data underscore that, prior to the current wave, GII.17 had never achieved sustained or widespread predominance in Brazil.

This ongoing resurgence of GII.17[P17] likely reflects a combination of antigenic novelty, immunological gaps in the post-pandemic population, and the emergence of polymerase type with improved viral fitness. Similar to the USA and European observations, our data indicate a decline in GII.4 circulation coinciding with the rise of GII.17[P17]. This genotype replacement phenomenon has been described previously for both GII.17 and GII.2 during periods when likely GII.4 population immunity was high^17,18,21^. Genomic analyses demonstrated that GII.17[P17] viruses that are currently prevailing in many countries, underwent a dynamic and adaptive evolutionary process characterized by recurrent and back-and-forth mutations and amino acid substitutions in the most external P2 subdomain of the VP1 capsid that resulted in enhanced binding to a broader range of host HBGA carbohydrates and antigenic diversification compared to earlier GII.17 clusters^52^. These combined structural and antigenic changes allowed the new GII.17 lineage to reach an optimally adapted phenotype and have facilitated its rapid spread across multiple regions.

This study has some limitations. The variability in the reporting of AGE cases, as well as the heterogeneity in surveillance capacity and reporting practices among different states likely contributed to uneven case detection. Furthermore, the analysis was limited to partial regions of the norovirus polymerase and capsid gene. More variable and immunologically relevant regions, such as the P2 subdomain within VP1, were not characterized, and amino acid changes within key antigenic epitopes were not assessed. Future studies are planned to include comprehensive characterization of the P2 subdomain and, where feasible, the complete capsid gene of the newly emergent GII.17[P17] to better understand its evolution over time and trace the origin of its introduction in Brazil.

In conclusion, our study, conducted between January 2023 and December 2024, documented the emergence and predominance of the GII.17[P17] genotype in Brazil, which displaced the previously dominant GII.4 Sydney[P16] strain. Notably, these genotypes displayed distinct age-distribution profiles: GII.17[P17] infected a broader range of age groups and disproportionately affected older children, adolescents and adults, whilst GII.4 Sydney[P16] predominantly affected children under five years of age. Genomic surveillance of noroviruses plays an important role in identifying shifts in genotype circulation, detecting the emergence of novel genotypes/variants, and characterizing evolutionary patterns that may reflect enhanced transmissibility and immune escape strains. Such information is essential for guiding vaccine design. Therefore, sustained, nationwide molecular surveillance remains crucial for monitoring future trends in the epidemiology of norovirus in Brazil.

## Methods

### Stool samples

This study included stool samples from medically attended patients (children and adults) collected between January 2023 and December 2024 with symptoms of AGE from ten states within three Brazilian regions: Southern (Rio Grande do Sul and Santa Catarina), Southeastern (Espírito Santo, Minas Gerais and Rio de Janeiro) and Northeastern (Alagoas, Bahia, Paraíba, Pernambuco and Sergipe). AGE was defined as sudden-onset diarrhea (≥ 3 liquid/semi-liquid evacuations in a 24-h period) that may be accompanied by fever, nausea, vomiting, or abdominal pain.

Diarrheic stool samples from patients with epidemiological records were collected by sentinels’ sites at States Central Laboratories and sent to the Laboratory of Comparative and Environmental Virology (LVCA) at Oswaldo Cruz Institute, Fiocruz. The LVCA houses the Rotavirus Regional Reference Laboratory (RRRL) and is part of the ongoing national network for rotavirus and norovirus surveillance coordinated by the General Coordination of Public Health Laboratories, Brazilian Ministry of Health. The surveillance is performed through a hierarchical network in which samples are provided by medical request in hospitals and health centers, monitored by the Brazilian Unified Health System (SUS).

### Ethics statements

This study is currently approved by the Ethics Committee of the Oswaldo Cruz Foundation (FIOCRUZ), Brazil (Approval number: CAAE 76063123.5.0000.5248) and was conducted according to the guidelines of the Declaration of Helsinki. Fecal samples were manipulated anonymously, and patients’ data were maintained securely. Laboratory activities performed are part of the public health surveillance tasks and Fiocruz Ethics Committee approved the waiver for informed consent.

### Viral RNA extraction

Viral nucleic acids were purified from 140 μL of clarified stool suspension (10% w/v) using the QIAamp Viral RNA Mini kit in an automated QIAcube platform (both from Qiagen, Valencia, CA, USA), according to the manufacturer’s instructions. Nucleic acids were eluted in 60 µl of the elution buffer AVE and were immediately stored at − 80 °C until the molecular analysis. In each extraction procedure, RNAse/DNAse-free water was used as negative control.

### Norovirus detection and quantification

Norovirus GI and GII were detected and quantified by using a TaqMan-based quantitative one step RT-PCR (RT-qPCR) duplex protocol with primers and probes targeting the ORF1/2 junction region^53,54^. Briefly, RT-qPCR reactions were performed with 5 µL of the extracted RNA in a final volume of 25 µL using the SuperScript^™^ III Platinum^™^ One-Step RT-qPCR Kit (ThermoFisher Scientific, Carlsbad, CA, USA) in the Applied Biosystems^®^ 7500 Real-Time PCR System (Applied Biosystems, Foster City, CA, USA). The thermal cycling conditions were carried out as follows: RT step at 50 °C for 60 min, an initial denaturation step at 95 °C for 5 min and 40 cycles of PCR amplification at 95 °C for 15 s and 60 °C for 1 min. All samples that crossed the threshold line showing a characteristic sigmoid curve with a cycle threshold (Ct) value <35 were regarded as positive. All runs included negative and non-template controls (NTC) to ensure the correct interpretation of the results throughout the study. To estimate norovirus viral load, a standard curve prepared by six 10-fold serial dilutions (10^6^–10^1^ genome copies (GC) per reaction) of a double-stranded DNA fragment (gBlock^®^ GeneFragment, Integrated DNA Technologies, Coralville, IA, USA) containing the norovirus amplification region sequence was used in each RT-qPCR reaction.

### Molecular characterization and genotyping

Norovirus-positive samples were subjected to conventional one-step RT-PCR for dual genotyping of polymerase and capsid regions. The reactions were performed using the Qiagen One Step RT-PCR kit (Qiagen) with primers Mon 432/G1SKR for GI and Mon 431/G2SKR for GII, that amplifies the ORF1/2 junction region, with expected PCR amplicon sizes of approximately 543 and 557 base pairs, respectively^55^. Purified amplicons were Sanger sequenced in both directions at the FIOCRUZ Institutional Sequencing Platform (PDTIS) on an ABI Prism 3730*xl* Genetic Analyzer (Applied Biosystems). Consensus sequences were obtained from chromatogram using Geneious Prime 2021.1.1 software (Biomatters Ltd., Auckland, New Zealand). Sequences were genotyped using the norovirus typing tools (https://mpf.rivm.nl/mpf/typingtool/norovirus/and/ and https://norovirus.ng.philab.cdc.gov). In addition, consensual sequences were confirmed in terms of closest homology sequence, using Basic Local Alignment Search Tool (BLAST).

### Phylogenetic analysis

Phylogenetic trees were constructed based in partial RdRp and VP1 protein (ORF1 and ORF2, respectively) using the neighbor-joining method for GI and GII norovirus. The best substitution models were selected based on the corrected Bayesian Information Criterion (BIC) value as implemented in MEGA v. 12 and model used in this study was Kimura 2-parameter (K2) + G in both regions (2000 bootstrap replications for branch support)^56^. Norovirus reference sequences were obtained from the National Center for Biotechnology Information (NCBI) database. Nucleotide sequences obtained in this study were submitted to NCBI GenBank (accession numbers: PX710099-PX710102; PX710104; PX710120-PX710149; PX710478-PX710494; PX710687-PX710696; PX710704; PX710912-PX710919; PX710948-PX710957; PX711237-PX711246; PX711252-PX711264; PX711272-PX711281; PX736900-PX736986; PX737092-PX737284 and PX737880).

### Statistical analysis

Statistical analyses were performed using GraphPad Prism v. 8.4.1 (GraphPad Software, San Diego,CA, USA). Box-and-whisker plots were produced to illustrate differences between medians within interquartile ranges. The Kolmogorov-Smirnov test was performed to assess the normal distribution of the data, and then the Mann-Whitney U test was used to compare viral load values ​​between genotypes. Chi-square and Fisher’s exact tests were used for analyzing categorical characteristics in contingency tables. For all analyses, a *p*-value < 0.05 was considered statistically significant. Furthermore, multiple component analysis (MCA) and uni/multivariate logistic regression were performed using R software version 4.5.3 (Vienna: R Foundation for Statistical Computing, 2026).

## Supplementary Information

Below is the link to the electronic supplementary material.

Supplementary Material 1

## Data Availability

The data that support the findings of this study are openly available in the GenBank database. The datasets generated and analyzed during the current study are available in the GenBank repository under the following accession numbers cited in the Methods section. This study is registered in the Brazilian National System for Genetic Heritage and Associated Traditional Knowledge Management (No. A837EB6). The datasets generated or analyzed during the current study are available in the GenBank repository (accession numbers: PX710099-PX710102; PX710104; PX710120-PX710149; PX710478-PX710494; PX710687-PX710696; PX710704; PX710912-PX710919; PX710948-PX710957; PX711237-PX711246; PX711252-PX711264; PX711272-PX711281; PX736900-PX736986; PX737092-PX737284 and PX737880).
