# Supplementary material for: Emergence and predominance of GII.17[P17] noroviruses in Brazil, 2023–2024
Source: Sci Rep. 2026 Apr 25;16:21955. doi: 10.1038/s41598-026-49730-6 (PMC13365198; doi:10.1038/s41598-026-49730-6)
Supplement: Supplementary file 1 — Supplementary Material 1 [file 41598_2026_49730_MOESM1_ESM.docx]

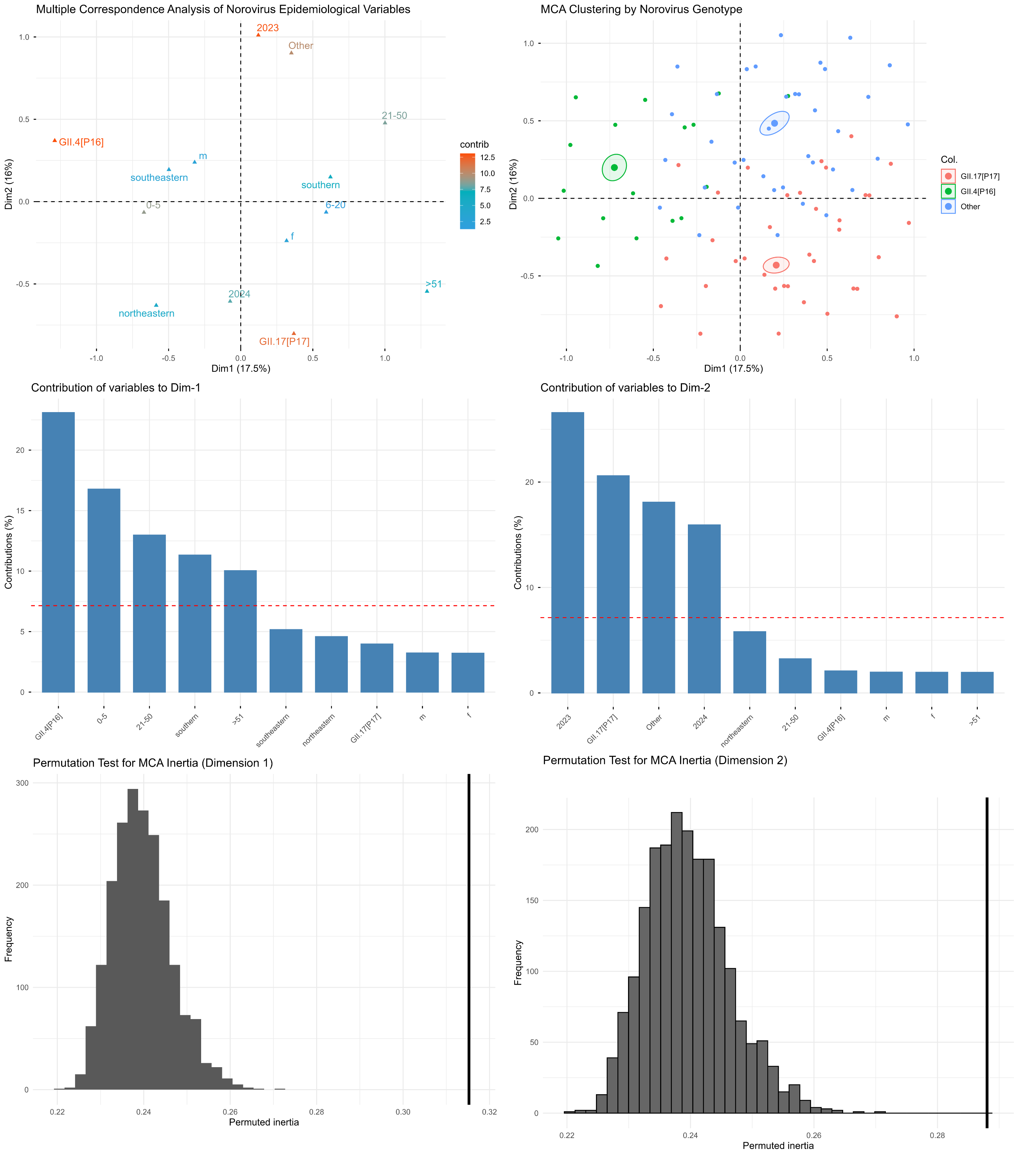


**F**

**E**

**C**

**D**

**B**

**A**

**Figure S1** - Multiple Correspondence Analysis (MCA) of norovirus epidemiological variables. **(A)** MCA factor map (Dimension 1 × Dimension 2) showing the position of the categories of the analyzed variables (age groups in years, region, period, and genotype). Labels indicate highlighted categories; the color scale indicates the relative contribution of the categories to the dimensions. **(B)** Map by norovirus genotype (GII.17[P17], GII.4[P16], and others), with confidence ellipses for the main clusters. **(C)** Bar chart with the percentage contribution of each variable to Dimension 1 (dashed red-line indicates expected average contribution)**. (D)** Bar chart with the percentage contribution of each variable to Dimension 2 (dashed line indicates expected average contribution). **(E)** Permutation test for the inertia of Dimension 1: histogram of the permuted inertia distribution and vertical line indicating the observed value (empirical inertia). **(F)** Permutation test for the inertia of Dimension 2, with the same representation.


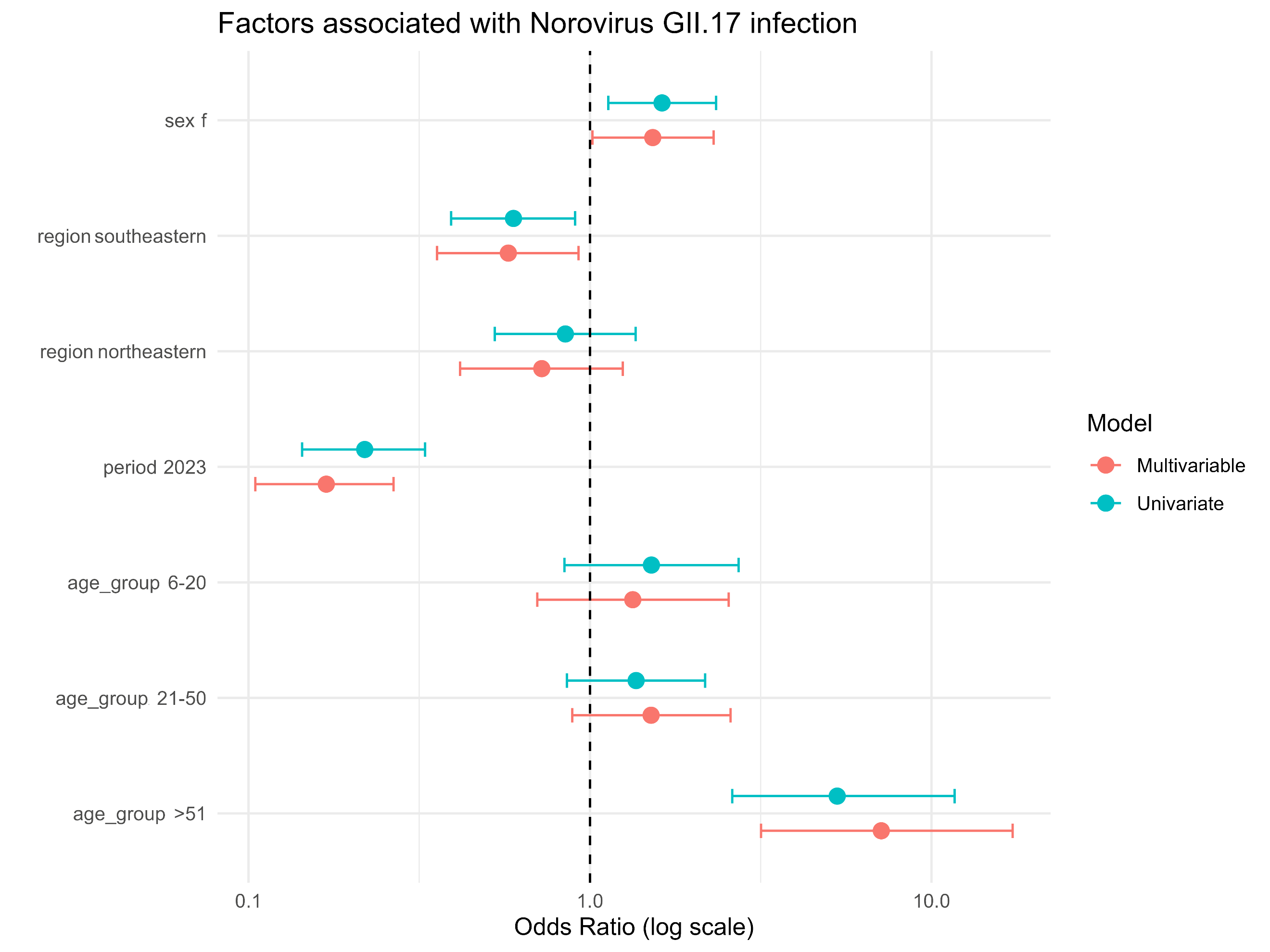


**Figure S2** - Factors associated with Norovirus GII.17 infection with odds ratios and 95% confidence interval (logarithmic scale). Forest plot comparing univariate (blue) and multivariate (orange) estimates for the variables: sex (female), region (southeastern, northeastern), period (2023), and age ranges in years (6–20, 21–50, >51). Horizontal lines represent 95% CI; the dashed vertical line indicates OR = 1 (no effect).


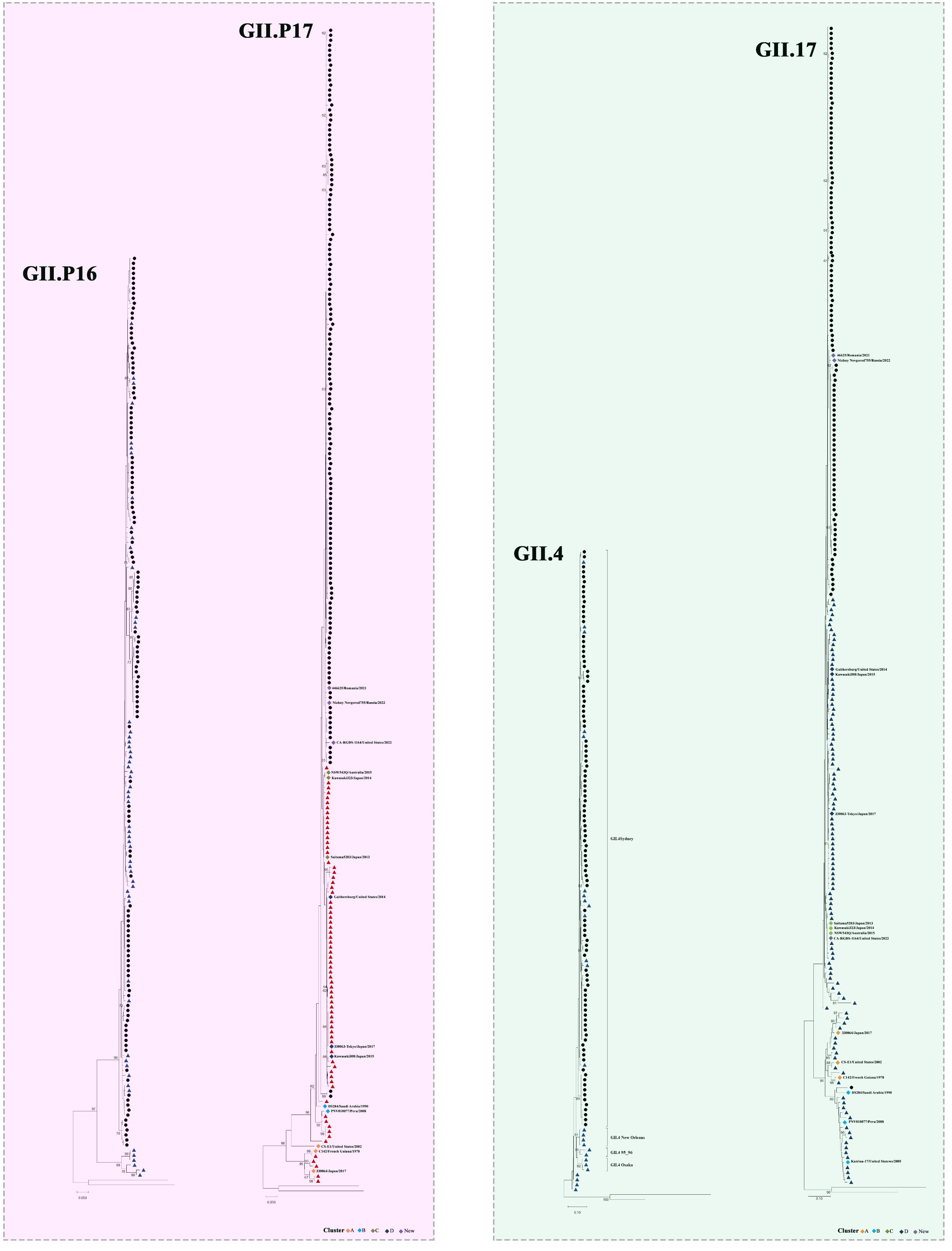


**B**

**A**

**Figure S3** Phylogenetic analyses based on RdRp (A) and VP1 (B) for noroviruses GII.P16 and GII.P17 (pink) as well as GII.4 and GII.17 (green). The samples obtained in this study were all marked with a black circle in both trees. The reference strains obtained for RdRp (A) were marked with a blue triangle (GII.P16) or a red triangle (GII.P17), while the prototypes of GII.P17 (cluster A, B, C, D and new) are shown in the figure. Downloaded reference strains (B) were marked with a blue triangle (for both GII.4 and GII.17) while the prototypes of GII.17 (cluster A, B, C, D and new) are shown in the figure. The neighbor-joining phylogenetic tree was constructed with bootstrap tests (2000 replicates), based on the Kimura two-parameter mode. Bootstrap values ​​above 60% are given at branch nodes.
